# Efficacy of Intravenous Lidocaine Versus Ketorolac for Acute Pain Relief in Patients With Suspected Renal Colic Presenting to the Emergency Department at Pakistan Ordnance Factories (POF) Hospital, Wah Cantt, Pakistan

**DOI:** 10.7759/cureus.113302

**Published:** 2026-07-24

**Authors:** Shadab Shadab, Sohaib Qureshi, Huma Hussain, Tazaeen Hina Kazmi, Sana Shafique

**Affiliations:** 1 Emergency Medicine, Cumberland Infirmary, North Cumbria Integrated Care (NCIC), NHS, Carlisle, GBR; 2 General Internal Medicine (GIM) and Gastroenterology, Cumberland Infirmary, North Cumbria Integrated Care (NCIC), NHS, Carlisle, GBR; 3 Accident and Emergency Medicine, Pakistan Ordnance Factories (POF) Hospital, Wah Cantt, PAK

**Keywords:** acute pain, emergency medicine, ketorolac, lidocaine, randomised controlled trial, renal colic

## Abstract

Background: Renal colic is a common urological emergency requiring prompt and effective analgesia. Although intravenous ketorolac is recommended as first-line therapy, its use may be limited in patients with contraindications to non-steroidal anti-inflammatory drugs (NSAIDs). Intravenous lidocaine has emerged as a potential non-opioid alternative. This study compared the analgesic efficacy and short-term safety of intravenous lidocaine with intravenous ketorolac in adults presenting with suspected renal colic.

Methods: This prospective, single-center, randomized controlled trial included 90 adults presenting to the emergency department with suspected renal colic and a Visual Analogue Scale (VAS) pain score of ≥7. Participants were randomly assigned to receive either intravenous lidocaine (1.5 mg/kg infused over 15 minutes; n = 45) or intravenous ketorolac (30 mg intravenous bolus; n = 45). The primary outcome was successful pain relief, defined as a reduction of at least three points on the VAS 30 minutes after treatment. Secondary outcomes included rescue analgesia requirements and adverse events.

Results: Successful pain relief was achieved in 31 patients (68.9%) in the lidocaine group and 27 patients (60.0%) in the ketorolac group (absolute risk difference 8.9%; 95% confidence interval, −10.8% to 28.6%; χ²(1) = 0.776, p = 0.378). Rescue analgesia was required in 14 patients (31.1%) receiving lidocaine and 18 patients (40.0%) receiving ketorolac. Adverse events were infrequent and mild, with transient dizziness reported in three patients receiving lidocaine and mild hypersensitivity reactions in two patients receiving ketorolac. No serious adverse events occurred.

Conclusions: Intravenous lidocaine provided analgesic efficacy comparable to intravenous ketorolac for the management of suspected renal colic, with a similar short-term safety profile. Intravenous lidocaine may be considered a useful alternative for patients in whom NSAIDs are contraindicated or poorly tolerated. Larger multicenter studies are warranted to confirm these findings.

## Introduction

Renal colic is one of the most common urological emergencies encountered in clinical practice and is most frequently caused by acute ureteric obstruction secondary to urinary calculi. Obstruction of the urinary tract leads to increased intraluminal pressure, ureteric smooth muscle spasm, and the release of inflammatory mediators, resulting in sudden onset of severe flank pain that may radiate to the groin. Patients commonly present with associated symptoms including hematuria, nausea, vomiting, dysuria, and urinary urgency. Owing to its intensity, renal colic is widely regarded as one of the most severe forms of acute pain and accounts for approximately 1% of emergency department (ED) visits worldwide [1,2].

The global lifetime prevalence of urolithiasis is estimated to range from 5% to 15%, with considerable geographical variation influenced by climate, dietary habits, metabolic disorders, and genetic predisposition [1,3]. The disease occurs more frequently in men than women, with a male-to-female ratio of approximately 2-3:1, and most commonly affects individuals between the third and fifth decades of life [3,4]. In South Asian countries, including Pakistan, the prevalence of urinary stone disease has been reported to be between 12% and 15%, representing a significant healthcare burden because of recurrent ED presentations and hospital admissions [4].

Rapid and effective analgesia remains the cornerstone of emergency management. Current international guidelines recommend non-steroidal anti-inflammatory drugs (NSAIDs) as first-line treatment because they inhibit cyclooxygenase-mediated prostaglandin synthesis, thereby reducing renal pelvic pressure, ureteric edema, and smooth muscle spasm [1,2,5]. Ketorolac is among the most commonly administered intravenous (IV) NSAIDs because of its rapid onset of action and well-established analgesic efficacy. However, NSAIDs may be contraindicated in patients with chronic kidney disease, gastrointestinal bleeding, peptic ulcer disease, uncontrolled hypertension, or significant cardiovascular disease. Furthermore, a proportion of patients continue to experience inadequate pain relief and require rescue analgesia despite standard NSAID therapy [5,6].

The increasing emphasis on opioid-sparing analgesia has stimulated interest in alternative non-opioid agents for the treatment of acute renal colic. IV lidocaine, traditionally used as a local anesthetic and class Ib antiarrhythmic agent, has demonstrated analgesic properties in neuropathic pain, postoperative pain, visceral pain syndromes, and selected ED presentations [7,8]. The proposed mechanism involves blockade of voltage-gated sodium channels, suppression of ectopic neuronal discharge, and modulation of central pain pathways, resulting in reduced pain perception [7,8].

Several clinical studies have evaluated IV lidocaine in patients presenting with renal colic. Soleimanpour et al. reported that IV lidocaine provided pain relief comparable to IV morphine while maintaining an acceptable safety profile [9]. Similar findings have been described by Sin et al., who demonstrated that IV lidocaine, either alone or in combination with ketorolac, is an effective non-opioid analgesic option for acute renal colic in the ED [10,11]. In addition, a systematic review by e Silva et al. concluded that IV lidocaine is both safe and effective for carefully selected patients presenting with acute pain in the emergency setting [12].

The use of IV lidocaine as an analgesic has evolved considerably over the past several decades. First described by Steinhaus and Howland in 1958 as an adjunct to nitrous oxide anesthesia, IV lidocaine was subsequently shown to possess analgesic and anesthetic-sparing properties beyond its established role as a local anesthetic and antiarrhythmic agent [13]. During the 1970s, pharmacokinetic and experimental studies further established its analgesic efficacy and provided the basis for safe therapeutic dosing [14,15]. Subsequent mechanistic studies and clinical trials supported its incorporation into multimodal perioperative analgesia, demonstrating reductions in postoperative pain, opioid consumption, and enhanced recovery, while international consensus guidelines have recommended standardized weight-based dosing with appropriate patient selection and monitoring [16,17]. More recently, systematic reviews and ED studies have demonstrated encouraging efficacy and safety of IV lidocaine as an opioid-sparing analgesic for acute painful conditions, including renal colic, using single bolus doses of 1-1.5 mg/kg administered over 10-15 minutes [10,18,19]. These findings provide the rationale for evaluating IV lidocaine as an alternative to ketorolac in patients presenting with suspected renal colic.

Despite these encouraging findings, evidence directly comparing IV lidocaine with IV ketorolac remains limited, particularly in low- and middle-income countries where NSAIDs continue to be the predominant first-line analgesic for renal colic. Establishing an effective alternative as a non-opioid analgesic is clinically important for patients in whom NSAIDs are contraindicated or poorly tolerated. Therefore, the present prospective randomized controlled trial was conducted to compare the efficacy and short-term safety of IV lidocaine with IV ketorolac in adult patients presenting with suspected renal colic to the ED.

## Materials and methods

Study design and setting

This prospective, single-center, randomized controlled trial was conducted in the Department of Accident and Emergency, Pakistan Ordnance Factories (POF) Hospital, Wah Cantt, Pakistan, over a six-month period from July 14, 2022, to December 31, 2022. The study was designed to compare the analgesic efficacy and short-term safety of IV lidocaine with IV ketorolac in adult patients presenting with suspected renal colic.

Ethical considerations

Ethical approval was obtained from the Institutional Ethical Review Committee of POF Hospital, Wah Cantt, prior to commencement of the study (Institutional Review Board (IRB) No. 4119/4/HOD ER/Hosp). Written informed consent was obtained from all participants before enrolment in accordance with institutional ethical requirements and the principles of the Declaration of Helsinki.

Participants

Adult patients presenting to the ED with clinically suspected renal colic were screened for eligibility. Patients aged between 18 and 65 years presenting with acute unilateral flank pain suggestive of renal colic and a Visual Analogue Scale (VAS) pain score of ≥7 were eligible for inclusion. Patients were excluded if they had a known allergy or hypersensitivity to lidocaine or ketorolac, hemodynamic instability, significant cardiovascular disease (including ischemic heart disease, previous myocardial infarction, atrial fibrillation, advanced heart block, Wolff-Parkinson-White syndrome, prolonged QT interval, or symptomatic bradycardia), chronic liver disease, chronic kidney disease, seizure disorder, inflammatory bowel disease, active gastrointestinal bleeding, hepatitis, altered mental status, pregnancy or breastfeeding, severe uncontrolled hypertension (blood pressure > 180/120 mmHg), or previous enrolment in the study.

Sample size

The sample size was determined prospectively during the study protocol development and IRB application. The calculation was performed using the WHO Sample Size Calculator based on an estimated population proportion of 15% for stone disease, as reported by Rizvi et al. [20], with an absolute precision of 8% and a 95% confidence level (α = 0.05). This yielded a required sample size of 90 participants. Accordingly, 90 patients were enrolled and randomized equally between the two treatment groups (45 participants per group).

Randomization, allocation concealment, and blinding

Eligible participants were enrolled consecutively and randomly allocated in a 1:1 ratio to receive either IV lidocaine or IV ketorolac using the lottery method. Allocation concealment was achieved using sequentially numbered, opaque, sealed envelopes prepared by the principal investigator before patient enrolment. Following written informed consent, the next sequential envelope was opened to determine treatment allocation. This was an open-label study; therefore, neither the participants nor the treating clinicians were blinded to the allocated intervention because of the differences in drug preparation and administration. Outcome assessment was performed according to the predefined study protocol.

Interventions

Participants allocated to the lidocaine group received IV lidocaine at a dose of 1.5 mg/kg diluted in 100 mL of 0.9% normal saline and administered over 15 minutes. This dosing regimen was selected based on the randomized controlled trial by Soleimanpour et al. [9], which demonstrated that IV lidocaine at this dose was effective and well tolerated for the management of acute renal colic. Participants allocated to the ketorolac group received a single 30 mg dose of IV ketorolac administered as a slow IV bolus. All patients were monitored throughout drug administration. Participants with persistent severe pain 30 minutes after treatment received rescue analgesia with IV nalbuphine (0.1 mg/kg) according to departmental protocol and remained under observation for a further 90 minutes to monitor for adverse events.

Outcome measures

The primary outcome was successful pain relief, defined as a reduction of at least three points on the VAS from baseline, assessed 30 minutes after administration of the study medication. Secondary outcomes included the occurrence of adverse drug reactions and the requirement for rescue analgesia during the observation period.

Statistical analysis

Statistical analysis was performed using IBM SPSS Statistics version 23.0 (IBM Corp., Armonk, NY, USA). Continuous variables were summarized as mean ± standard deviation (SD), whereas categorical variables were presented as frequencies and percentages. Baseline demographic characteristics were compared descriptively between the treatment groups.

The primary outcome (successful pain relief) and categorical secondary outcomes (rescue analgesia requirement and adverse events) were compared using the Pearson Chi-squared test. Fisher's exact test was used when expected cell counts were small. Effect estimates are presented as absolute risk difference, relative risk, 95% confidence intervals (CIs), and Phi coefficient as the measure of effect size for Chi-squared analyses.

All statistical tests were two-sided, and a p-value < 0.05 was considered statistically significant. Outcome data were complete for all randomized participants. Therefore, an intention-to-treat analysis was performed, with all randomized participants analyzed in the groups to which they were originally allocated. 

## Results

Participant flow

**Figure 1:**
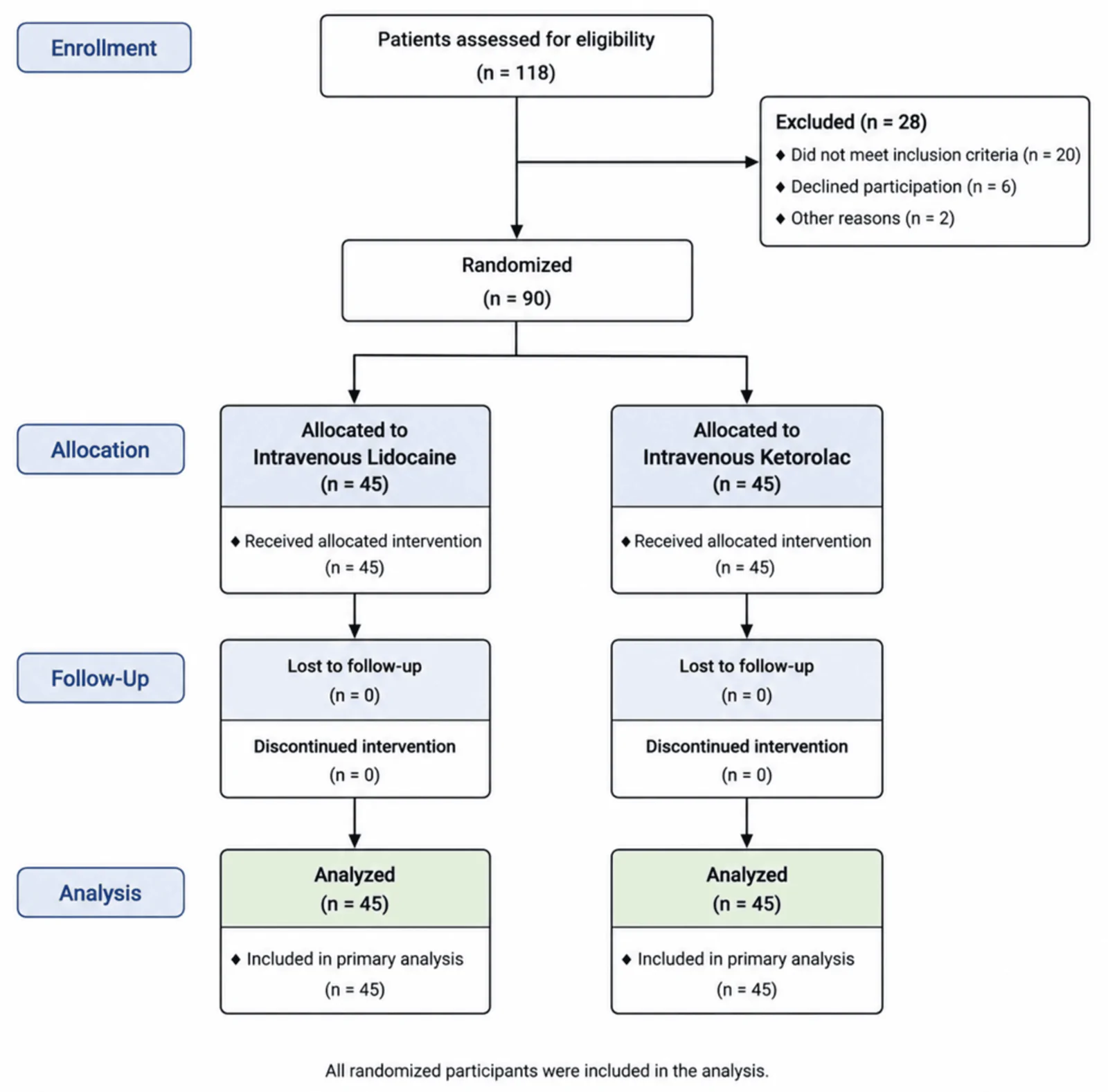
CONSORT flow diagram CONSORT: CONsolidated Standards Of Reporting Trials

A total of 90 patients with clinically suspected renal colic were enrolled and randomized between July and December 2022. Forty-five participants were allocated to receive IV lidocaine and 45 to receive IV ketorolac. All randomized participants received the allocated intervention, completed the study, and were included in the final analysis. No participants were lost to follow-up, withdrawn, or excluded after randomization; therefore, outcome data were available for all participants. The participant flow through the study is illustrated in Figure 1.

Baseline characteristics

The baseline demographic and clinical characteristics of the study participants were comparable between the two treatment groups (Table 1). The mean age was 42.2 ± 15.2 years in the IV lidocaine group and 41.6 ± 14.6 years in the IV ketorolac group. Most participants were between 19 and 50 years of age (60.0% vs. 68.9%, respectively). There were 24 men (53.3%) and 21 women (46.7%) in the lidocaine group compared with 27 men (60.0%) and 18 women (40.0%) in the ketorolac group. Diabetes mellitus was present in 10 patients (22.2%) receiving lidocaine and six patients (13.3%) receiving ketorolac, while hypertension was reported in 10 (22.2%) and 17 (37.8%) patients, respectively.

**Table 1:** Baseline demographic and clinical characteristics of the study participants SD: standard deviation

Characteristic	Intravenous lidocaine (n = 45)	Intravenous ketorolac (n = 45)
Age (years), mean ± SD	42.2 ± 15.2	41.6 ± 14.6
Age 19-50 years, n (%)	27 (60.0)	31 (68.9)
Age 51-64 years, n (%)	18 (40.0)	14 (31.1)
Male, n (%)	24 (53.3)	27 (60.0)
Female, n (%)	21 (46.7)	18 (40.0)
Diabetes mellitus, n (%)	10 (22.2)	6 (13.3)
Hypertension, n (%)	10 (22.2)	17 (37.8)

Primary outcome

The primary outcome was successful pain relief, defined as a reduction of at least three points on the VAS from baseline, assessed 30 minutes after administration of the study medication. Successful pain relief was achieved in 31 of 45 patients (68.9%) in the IV lidocaine group compared with 27 of 45 patients (60.0%) in the IV ketorolac group (Table 2). The absolute risk difference was 8.9% (95% CI: −10.8% to 28.6%). The relative risk of treatment success with IV lidocaine compared with IV ketorolac was 1.15 (95% CI: 0.84-1.56). The difference between the treatment groups was not statistically significant (χ²(1) = 0.776, p = 0.378, φ = 0.093), indicating a small effect size.

**Table 2:** Comparison of treatment outcomes between the intravenous lidocaine and intravenous ketorolac groups CI: confidence interval

Outcome	Intravenous lidocaine (n = 45)	Intravenous ketorolac (n = 45)	Statistic
Successful pain relief, n (%)	31 (68.9)	27 (60.0)	χ²(1) = 0.776, p = 0.378
Unsuccessful pain relief, n (%)	14 (31.1)	18 (40.0)	
Absolute risk difference (95% CI)	8.9%		−10.8% to 28.6%
Relative risk (95% CI)	1.15		0.84-1.56
Effect size (Phi coefficient, φ)			0.093

Secondary outcomes

Rescue Analgesia

Rescue analgesia with IV nalbuphine was administered to participants who did not achieve the predefined criterion for successful pain relief at 30 minutes. Consequently, 14 patients (31.1%) in the IV lidocaine group and 18 patients (40.0%) in the IV ketorolac group required rescue analgesia. Consistent with the primary outcome analysis, the difference between groups was not statistically significant (χ²(1) = 0.776, p = 0.378, φ = 0.093).

Adverse Events

Both study medications were generally well tolerated (Table 3). Three patients (6.7%) in the IV lidocaine group experienced transient dizziness during drug administration, which was self-limiting and did not require any intervention. Two patients (4.4%) in the IV ketorolac group developed mild hypersensitivity reactions that resolved following treatment with IV pheniramine. No serious adverse events, treatment discontinuations, or deaths occurred in either treatment group. The overall incidence of adverse events did not differ significantly between groups (Fisher's exact test, p = 1.000).

**Table 3:** Adverse events ^†^Fisher's exact test.

Outcome	Lidocaine (n = 45)	Ketorolac (n = 45)	p-value
Any adverse event, n (%)	3 (6.7)	2 (4.4)	1.000^†^
Transient dizziness, n (%)	3 (6.7)	0	-
Mild hypersensitivity reaction, n (%)	0	2 (4.4)	-
Serious adverse events, n (%)	0	0	-
Treatment discontinued, n (%)	0	0	-

Outcome completeness

Outcome data were available for all randomized participants. There were no missing data, and all analyses were performed on the complete study population according to their allocated treatment groups.

## Discussion

This prospective randomized controlled trial compared the analgesic efficacy of IV lidocaine with IV ketorolac in adult patients presenting to the ED with suspected renal colic. The study demonstrated that IV lidocaine achieved successful pain relief in a higher proportion of patients than IV ketorolac (68.9% vs. 60.0%); however, the difference was not statistically significant (p = 0.378). These findings suggest that IV lidocaine provides analgesia comparable to ketorolac and may be considered a reasonable alternative in appropriately selected patients.

Renal colic is one of the most painful conditions encountered in emergency medicine, and prompt pain relief remains the primary objective of management [1,2]. Current international guidelines recommend NSAIDs as first-line therapy because they reduce prostaglandin synthesis, thereby decreasing ureteric edema, smooth muscle spasm, and renal pelvic pressure [1,2,5]. Ketorolac is widely used because of its rapid onset of action and favorable efficacy profile. Nevertheless, NSAIDs are unsuitable for some patients because of contraindications such as chronic kidney disease, gastrointestinal bleeding, peptic ulcer disease, or cardiovascular disorders [5,6]. Identifying effective non-opioid alternatives is therefore of considerable clinical importance.

The findings of the present study are consistent with previous reports evaluating IV lidocaine for renal colic. Soleimanpour et al. demonstrated that IV lidocaine provided analgesia comparable to IV morphine and significantly reduced pain in patients presenting with renal colic [9]. Similarly, Sin et al. reported favorable analgesic outcomes following IV lidocaine administration in the ED [10]. More recently, Motov et al. observed that IV lidocaine, either alone or in combination with ketorolac, may be an effective opioid-sparing strategy for the management of suspected renal colic [11]. Our findings support these observations and suggest that IV lidocaine can provide clinically meaningful pain relief comparable to standard NSAID therapy.

The analgesic effects of IV lidocaine are believed to result from blockade of voltage-gated sodium channels, suppression of ectopic neuronal activity, and modulation of central pain pathways [7,8]. Unlike NSAIDs, lidocaine does not inhibit prostaglandin synthesis and therefore may represent a useful treatment option for patients in whom NSAIDs are contraindicated. Furthermore, IV lidocaine has demonstrated efficacy in a variety of acute and chronic pain conditions, supporting its role as a non-opioid analgesic in carefully selected patients [7,8,12].

Both treatment regimens were generally well tolerated in the present study. Three patients receiving IV lidocaine experienced transient dizziness, while two patients treated with ketorolac developed mild hypersensitivity reactions that resolved with standard treatment. No serious adverse events were observed in either group. Although the study was not powered to detect differences in safety outcomes, these findings are consistent with previous reports demonstrating that IV lidocaine has an acceptable safety profile when administered at recommended doses with appropriate patient selection and monitoring [9,12].

The present study has several strengths. It employed a prospective randomized controlled design, enrolled patients using clearly defined eligibility criteria, and compared two commonly used non-opioid analgesic strategies in a real-world ED setting. The standardized treatment protocol also reduced variability in patient management, thereby improving the reliability of the findings.

Several limitations should also be acknowledged. First, this was a single-center study with a relatively small sample size, which may have limited the statistical power to detect modest differences between treatment groups. Second, detailed serial pain scores beyond the primary outcome were unavailable, preventing a more comprehensive analysis of pain reduction over time. Third, patients were observed only during their ED stay, and long-term outcomes, recurrence of pain, and patient satisfaction were not evaluated. Finally, although randomization reduced selection bias, the absence of blinding may have introduced the potential for observer bias.

Future multicenter randomized controlled trials with larger sample sizes, standardized serial pain assessments, and longer follow-up are required to further establish the comparative efficacy and safety of IV lidocaine in patients with acute renal colic. Studies evaluating patient-reported outcomes, cost-effectiveness, and the role of IV lidocaine in patients with contraindications to NSAIDs would also provide valuable evidence for clinical practice.

Overall, the findings of this study suggest that IV lidocaine offers analgesic efficacy comparable to IV ketorolac for the management of suspected renal colic in the ED. Given its favorable short-term safety profile and potential role as an opioid- and NSAID-sparing analgesic, IV lidocaine may be considered a useful alternative in appropriately selected patients.

Study limitations

This study has several limitations. First, the sample size was determined pragmatically during the study design phase and was not based on a formal a priori calculation using predefined assumptions regarding effect size, significance level, and statistical power. Consequently, the study may have been underpowered to detect modest differences between the treatment groups and was not designed to demonstrate equivalence or non-inferiority between IV lidocaine and ketorolac. Therefore, the absence of a statistically significant difference should not be interpreted as evidence of equivalent efficacy.

Second, the diagnosis of renal colic was based on the patients' clinical presentation and the treating physician's clinical assessment in the ED. Although patients subsequently underwent imaging investigations, including CT kidneys, ureters, and bladder (KUB) or ultrasonography as clinically indicated, these follow-up data were not collected as part of the study. Consequently, some participants may not have had radiologically confirmed renal colic.

Finally, patients with contraindications to NSAIDs or IV lidocaine, including those meeting our predefined exclusion criteria, were excluded from the study. As a result, the findings may not be generalizable to these patient populations, and the prevalence of such subgroups could not be determined. Future adequately powered multicenter randomized controlled trials with standardized diagnostic confirmation and inclusion of patients with contraindications to NSAIDs are warranted to validate and extend these findings.

## Conclusions

In this randomized controlled study, no statistically significant difference was observed between IV lidocaine and IV ketorolac in the management of acute pain in adult patients presenting to the ED with suspected renal colic. Both medications were well tolerated, with only mild, self-limiting adverse events reported. These findings suggest that IV lidocaine may represent a potential non-opioid analgesic alternative, particularly for patients in whom NSAIDs are contraindicated or poorly tolerated. However, given the study's methodological limitations, including the absence of a formal a priori sample size calculation and the lack of imaging confirmation in all participants, these findings should be interpreted with caution. Larger, adequately powered multicenter randomized controlled trials are required to confirm the efficacy and safety of IV lidocaine in this setting.
